# Epidemiologic characteristics, knowledge and risk factors of unintentional burns in rural children in Zunyi, Southwest China

**DOI:** 10.1038/srep35445

**Published:** 2016-10-17

**Authors:** Shangpeng Shi, Huajun Yang, Ya Hui, Xiang Zhou, Tao Wang, Ya Luo, Huiyun Xiang, Xiuquan Shi

**Affiliations:** 1 Department of Epidemiology and Health Statistics, School of Public Health, Zunyi Medical University, Zunyi, Guizhou, China; 2 Third Affiliated Hospital of Zunyi Medical University, Zunyi, Guizhou, China; 3 Center for Injury Research and Policy, The Research Institute at Nationwide Children’s Hospital, The Ohio State University College of Medicine, Columbus, OH, USA; 4 Center for Pediatric Trauma Research, The Research Institute at Nationwide Children’s Hospital, The Ohio State University College of Medicine, Columbus, OH, USA

## Abstract

We investigated the knowledge level and risk factors for pediatric unintentional burns in rural Southwest China with an aim to provide basic evidence for the prevention strategies. A stratified sampling method was used to recruit 1842 rural children from 9 schools. Self-reported burns during the past 12 months and relevant risk factors were collected by questionnaires. The burn incidence of all surveyed children was 12.7% (95% confidence interval [95% CI] 11.2–14.2%). We found that burn incidence had a trend to increase with the increasing school grade level and a trend to decrease with increasing knowledge scores on burns. The top two causes of burns were hot liquids (36.3%) and hot object (29.5%). More than 30% of children had little knowledge about preventive measures and how to give first-aid after burns. The main risk factors for burns included female gender, left-behind children by parents who were working in cities, and poor mother school education level. As the incidence of pediatric unintentional burns was high in rural southwest China, schools, families, and local public health agencies should put efforts into health education targeting burn prevention and first-aid measures after burns, particularly in “left-behind” children and those with mothers with poor education.

Unintentional injuries in childhood are serious public health problems in modern society. They not only result in great medical costs and economic loss, but also cause immeasurable psychological consequences in children themselves and their families^1^. Annually, over 875,000 children less than 18 years old die around the world as a result of injuries, and 80% of these injuries occur in low- and middle-income countries (LMICs)^2^,^3^. The direct health cost of injury was estimated to be at least 44 billion RMB (equivalent to US $5.4 billion) each year^4^. For fire-related deaths, the rate in low-income countries was close to 11 times higher than that in high-income countries. The WHO Global Burden of Disease estimates there were more than 7.1 million fire-related unintentional burns in 2004, which was an overall incidence rate of 110 per 100,000 per year. In addition, 49% of affected children suffered some form of disability after a burn, with 8% being left with a permanent physical disability^5^,^6^.

Pediatric unintentional burns are the world’s most prominent of all unintentional injuries. Pediatric unintentional burns are the third most frequent cause of injury resulting in death, surpassed only by motor vehicle crashes and falls. Survivors of severe burns may require continuing care and suffer from severe long-term consequences in physical, psychological and social dimensions, especially when specific body areas (e.g. face, hands or legs) are affected^7^,^8^,^9^. Previous reports have shown that burns are a major cause of morbidity and mortality worldwide, especially in LMICs^10^,^11^.

As the most populous developing country, China has an annual incidence of injuries of 16.1~21.9% for all ages^12^. Incidence of pediatric unintentional burns ranges from 2.9% to 20.6% in China. Some studies reported a higher incidence in rural areas than in urban areas^13^,^14^, while a few reported a higher incidence in urban areas^1^,^15^. Pediatric unintentional burns have become a common public health issue in China.

In the past several decades, many studies have investigated pediatric burns in rural areas in China. One study reported that dangerous farm environments and lack of adult supervision put farm children at high risk of unintentional burns^16^. However, the authors only compared the “left-behind” children (whose parents were away in cities, while their children were left behind to be cared for by a single parent or other family members) and those children living with two parents^16^. Another study conducted in rural Zunyi, Southwest China focused on the assessment of knowledge and attitude improvement about unintentional injuries among school-aged children, but burns only accounted for a small part in this study^17^. Some risk factors for pediatric unintentional burns may be common in rural and urban children^13^,^14^,^15^,^18^, while some factors might be unique to rural children. However, little work has focused on pediatric unintentional burns in low-income rural areas of China.

This study was designed to investigate pediatric unintentional burns in a rural area of China. We described epidemiological characteristics of burns, identified risk factors, and assessed children’s prevention and first-aid knowledge with an aim to build evidence for future intervention programs to prevent unintentional burns in the rural pediatric population in China.

## Methods

### Survey sites and sampling

Zunyi city is located in the Guizhou province, southwest China, and has jurisdiction over 3 districts and 12 counties. The city covers 11,882 square miles and the majority of areas are typical mountain areas. It has a total population of approximately 7.5 million and about 6.3 million rural residents. The 2010 population census in this area counted about 2.0 million children under age 18, accounting for 27.0% of the total population^12^.

A multistage cluster sampling method was used in this study. All respondents were selected from a pool of school students. First, we randomly sampled 3 counties: Yuqing, Fenggang, and Tongzi counties. Then we randomly selected 9 schools (3 middle schools and 6 primary schools) from 5 towns in the 3 counties. All students aged 7 to 17 years in school grades 3 through 7 were included. The average age of grades 3-4 (combined due to small sample size), grade 5, grade 6 and grade 7 was (10.11 ± 0.74), (11.28 ± 0.75), (12.29 ± 0.71) and (13.36 ± 0.74), respectively. Students were asked to participate if they were at school during the survey period from November 2014 to June 2015.

### Definition of burns

In our survey, a burn injury was defined using one of the following three criteria: (1) the child suffered a burn and was treated by parents or school teachers; (2) the child was diagnosed to have a burn-related injury by a doctor at a clinic or a hospital; (3) the child was absent from school or rested for half a day or longer because of a burn. Burn injuries suffered up to 12 months prior to the interview were included^19^.

### Sample size estimation

Sample size was calculated for a cross-sectional survey. The initial sample size of 1,068 was estimated using the formula^20^ *N* = *Zα*^2^ × *p* × *q*/(d^2^), where α (alpha) was probability of type I error, p × q was the maximum possible estimate of variance, and d (delta, δ) was the permissible error. Considering missing answers in the questionnaire and non-response, we added 20% to the estimated sample so the final estimated sample size needed for our study was 1282.

### Data collection and quality control

Data were collected at each school using our survey questionnaire. Trained interviewers entered each classroom, explained the purpose of the survey, and distributed the paper questionnaires. Questionnaires asked about (1) demographics, including children’s age, gender, school grade level, whether they are a single child in the family, and whether they attended boarding school or not; (2) family living arrangement (we classified family status into 4 subgroups: living with both parents, living with father only, living with mother only, and living with someone other than parents. Children in the last 3 groups were classified as left-behind children^16^); (3) mother’s educational level and father’s educational level (data from the school records); (4) family income status (because it was difficult to get accurate data about family income, our study asked children to self-report their family economic status as poor, middle, or high. In addition, we designed several questions such as “Parents’ occupations”, “Do your parents often give pocket money”? to assess the family socio-economic status); (5) Child knowledge and practices about burn events and first-aid/medical treatments after burns (Questions were designed using relevant previous studies by Peck *et al.*^21^, Khandarmaa *et al.*^15^ and Davies *et al.*^22^).

The outcome variable in our study was whether a child had unintentional burns during the 12 months prior to the survey. For each reported burn event, the child self-reported the cause of burn, time and season of burn event, place of burn and body part affected. Because this was a self-reported survey study, total body surface burn area was not asked due to the challenge in determining it in a survey.

Interviewers and teachers were present in the classrooms during the interview to help children understand the questions if necessary. When students submitted their questionnaires, interviewers were required to check every questionnaire to make sure each survey question was answered. If not, the questionnaire was returned to the student to provide the missing answers.

### Ethics statement

The study protocol was approved by the 9 participating schools and Institutional Review Board of Zunyi Medical University (No. ZYMEC 2013-001). Research was carried out in accordance with the Helsinki Declaration. A letter was sent to the students’ parents or legal guardian(s) to explain the aims, study procedures, and data confidentiality assurance. All guardians and participants provided written informed consent before the survey was conducted.

### Statistical analysis

A database was constructed using the EpiData 3.1 (http://www.epidata.dk). Data were double-entered to reduce errors. Analysis was performed using the statistical software package SPSS v18.0 (SPSS Inc., Chicago, IL, USA). Statistical results were reported with number (percentage) and mean ± SD (standard deviation). Risk factors for burn were identified using a bivariate logistic regression analysis. All variables that met statistical significance level of *P-*value 0.05 in bivariate analysis were examined in the unconditional multivariate logistic regression. All statistical significance tests were two tailed and *P*-value < 0.05 or 95% CI of OR not containing 1 were considered statistically significant.

## Results

### General characteristics of survey population and unintentional burns

A total of 1842 children (mean age 11.9 ± 1.4 years) were interviewed (response rate 99.3%, 1842/1855) in 9 schools. Of those interviewed students, 54.1% (997/1842) were males and 45.9% (845/1842) were females. Overall, 234 children (12.7%, [95% CI] 11.2–14.2%) reported their unintentional burns that occurred in the 12 months prior to the survey.

As we have hypothesized, most burns were caused by hot liquid (36.3%), hot objects (29.5%) and fire (25.2%). The leading injured body parts were hands or arms. Burns to limbs accounted for more than three-quarters of the total burns, while head and trunk accounted for less than one-quarter. Home was the most frequent place for pediatric burns, where 91.0% of burns occurred. The main activity when the burn event happened was when the child was doing house chores (42.7%). In terms of time of burns, most burns (60.2%) occurred at noon (about lunch time in a day). Pediatric burns most commonly occurred in summer (37.6%) and least in spring (16.2%) (Table 1).

### Burn incidence among children by grade, knowledge level and gender

The overall incidence was higher in females than males (15.7% vs. 10.1%, *P* < 0.001). For students in grades 3–4, 5 and 7, the incidence of burns did not differ between females and males. However, for children in grade 6, the incidence was higher in females than males (17.9% vs. 8.8%, *P* = 0.002). Total burn incidence showed a trend to increase with increasing grade level (Cochran-Armitage trend test, *P* = 0.003), but this trend only appeared in females.

A burn knowledge score was created for each child using a total of eight single-choice questions based on previous studies by Peck *et al.*^21^, Khandarmaa *et al.*^15^ and Davies *et al.*^22^. Each correct answer would award one score. The highest score was eight, which meant all questions were answered correctly. For both males and females, incidence of unintentional burns showed a trend to decrease with increasing burn knowledge scores (Cochran-Armitage trend test, *P* < 0.001, Table 2).

### General knowledge about burn prevention and first-aid

Results in Table 3 suggested that most (95.9%) children had prevention knowledge about the proper use of a pressure cooker. With regards to setting off firecrackers and making an emergency call after a burn event, these two questions were answered correctly by 91.4% and 91.3% of surveyed students, respectively. The question about proper safety when seeing fallen wires on the road was answered correctly by 91.2% of students. However, more than 30% of interviewed children had little knowledge about how to give first-aid after burns. More than one fifth of children had little idea about how to proceed when a fire happens, and nearly 15% of children did not answer correctly the question of what should not do when a fire happens. The survey question about electric shock was not answered correctly by more than a quarter of children (Table 3).

### Risk factors of burns

Table 4 provides results of bivariate analysis. We identified 7 potential risk factors for burns. Children in grades 3–4 and 5 had a significantly higher risk of burns compared with grade 7. Gender was found to play a significant role in burns. Compared with males, females were at a significantly higher risk of burn injury (OR = 1.66). Children who were left-behind (OR = 1.44) were significantly more likely to suffer burns compared with children who were living with two parents. Additionally, children whose parents had lower education (less than secondary school) were significantly more likely to suffer burns. Compared with children who were living at home, the boarding children were at a significantly higher risk of burn injuries (OR = 1.54). When compared with middle class families, children living in high income families were less likely to sustain burns (OR = 0.63).

Results from multivariate logistic regression analyses revealed 4 risk factors that were associated with risk of burns, including grades 3-4 (OR = 1.84, [95% CI] 1.20–2.80), grade 5 (OR = 2.00, [95% CI] 1.33–2.99), females (OR = 1.70, [95% CI] 1.29–2.25), being “left-behind” children (OR = 1.35, [95% CI] 1.02–1.80), mother education level at primary school or lower (OR = 2.43, [95% CI] 1.61–3.69).

## Discussion

To our knowledge, this study was the largest sample of a population-based survey on pediatric unintentional burns in rural southwest China. Our findings were consistent with previous epidemiological surveys, which suggested that leading causes of pediatric burns were hot liquids and objects^23^,^24^; and the upper extremities (hands/arms) were the most injured body parts^13^,^24^. Previous studies reported that pediatric unintentional burns occur most frequently at home^2^,^6^,^9^. In our study, 37.6% of unintentional burns occurred in summer, which is slightly higher than the Karimi *et al.* study (32.2%)^25^. In addition, we found that pediatric burns commonly occurred when the child was doing house chores. Plausible explanations for high risk of pediatric unintentional burns in rural areas of China include: rural house construction most commonly consisting of only one or two rooms, making it difficult to effectively prevent children from burn hazards associated with cooking; children usually help their families in different household chores; high burn risk house chores of cooking, splashing hot liquids and touching hot objects. It is a common practice for children to cook meals at noon when parents are busy doing farm work in rural areas of Zunyi, which puts children at high risk of burns when they are not supervised by adults.

Pediatric injuries affect low- and middle-income countries more than high-income countries^6^. Our finding of 12.7% incidence of pediatric unintentional burns in Zunyi is higher than in many countries. In the United States, the crude rate of burns was reported as 0.16% in children^26^. One study from Pakistan (economically comparable to China) reported a rate of 9% for pediatric burns^2^. The incidence of pediatric burns in our study was also higher than in some other regions of China, such as Pucheng, Shaanxi province where a burn incidence of 2.9% was reported in a recent study^18^. The higher incidence found in rural southwest China in our study than in other regions might be due to living environment of the rural people in Zunyi. Residents in rural areas of Zunyi often cook their food in an open place, exposing children to flames, hot liquids or other heat sources; therefore children are at high risk of burns. Families in rural areas of Zunyi usually do not have a separate kitchen, which is another risk factor for pediatric burns.

Studies that examined gender differences in pediatric burns reported inconsistent results. Some studies reported a higher burn incidence in males than in females^9^,^27^. Our study found that the overall incidence was higher in females than in males, which is consistent with a few other studies^28^,^29^. One plausible explanation is that females in rural areas of Zunyi are more likely to do house chores than males^30^; therefore, females are at a higher risk of burns than males.

We found that the higher the child’s knowledge about burn prevention and first-aid treatments, the lower the incidence of pediatric unintentional burns. Our results suggested that more educational programs to increase awareness about burns may be required to reduce risk of pediatric unintentional burns in rural areas of Zunyi. However, our result found that over 30% of children did not have correct knowledge about how to provide first-aid after burn events. They reported wrong ways to treat burns themselves, such as spreading alcohol and using toothpaste. Such self-treatments are likely to cause infections or to prolong the healing process. We also found that many children did not provide correct answers about fire and electric shock. By and large, lack of appropriate knowledge of burn prevention and first-aid treatments are the plausible reasons for many pediatric unintentional burns in rural areas of China^31^. Our results underscore the importance to raise awareness about pediatric unintentional burns and first-aid treatments among caregivers and children in rural areas of China. Previous research reported school-family-individual (SFI) multi-level education intervention^17^ as an effective approach to improve burns knowledge among rural children and to reduce the incidence of pediatric unintentional burns.

“Left-behind” children is a unique social problem in modern rural China that puts millions of children at high risk for unintentional injuries. Our finding of a significantly higher burn incidence in “left-behind” children than children who lived with both parents is consistent with reports by Shi *et al.*^12^ and Shen *et al.*^16^. A study in Kurdish children^11^ reported similar findings. Adult supervision plays an important role in pediatric burn prevention^14^,^31^. “Left-behind” children were at significantly higher risk of burns than other children in our study maybe because of lack of appropriate adult supervision. Mother’s low school education was also a significant risk factor for burns in our study. Our finding was similar to a study by Peck^26^ that reported low mother’s school education was associated with a higher risk for pediatric unintentional burns around the world.

Due to the fact that our study is a cross-sectional survey, there are several limitations. First, the outcome measure (whether the student suffered a burn or not) relied solely on self-reported data, which might underestimate the problem because some minor burns might be forgotten. Second, we collected data about burns suffered in the 12 months prior to the survey, but we could not verify the accuracy of reported burn events. Finally, our survey was conducted among school-aged children in grades 3 to 7 in rural schools of Zunyi, therefore, our results may not be generalizable to all children in southwest China, such as preschool children, children in urban cities, those with disabilities who could not attend school, and those children who dropped out the school. Future research should consider studying burn injury risk among adolescents and preschool children in rural areas.

## Conclusions

Despite some limitations, our study provides some preliminary evidence about an important pediatric injury issue in China. Unintentional burns appear to be a major health problem in children in rural areas of Zunyi in southwest China. Burn prevention programs should target high risk population groups such as “left-behind” children, and children whose mothers have poor school education.

## Additional Information

**How to cite this article**: Shi, S. *et al.* Epidemiologic characteristics, knowledge and risk factors of unintentional burns in rural children in Zunyi, Southwest China. *Sci. Rep.* **6**, 35445; doi: 10.1038/srep35445 (2016).

## Figures and Tables

**Table 1:** Characteristics of unintentional burns among school-aged rural children, in Zunyi, People’s Republic of China.

Burn events	N (234)	%	Burn events	N (234)	%
Cause			Activity when burned		
Hot liquid	85	36.3	Doing house chore	100	42.7
Hot objects	69	29.5	Eating	45	19.2
Fire	59	25.2	Playing	42	17.9
Hot food	21	9.0	Other	47	20.2
Affected body part			Burn event time		
Hands/arms	147	62.8	Noon	141	60.2
Feet/leg	55	23.5	Morning	35	15.0
Head	19	8.1	Afternoon	30	12.8
Trunk	13	5.6	Evening	28	12.0
Burn event location			Season (months)		
Home	213	91.0	Spring (2,3,4)	38	16.2
Public place	13	5.6	Summer(5,6,7)	88	37.6
School	3	1.3	Autumn(8,9,10)	39	16.7
Other	5	2.1	Winter(11,12,1)	69	29.5

**Table 2:** Burn incidence among school-aged rural children by school grade, burn knowledge scores, and gender, Zunyi, China.

Variables	Male			Female		Total burn incidence (%)	*P*
	# of student reported burn/Total students	Burns Incidence (%)	# of student reported burn/Total students	Burns Incidence (%)			
Grade level						0.003
3-4	19/197§	9.6	17/161*	10.6	10.1Δ	0.775
5	17/229	7.4	23/193	11.9	9.5	0.116
6	25/283	8.8	41/229	17.9	12.9	0.002
7	40/288	13.9	52/262	19.8	16.7	0.061
Knowledge scores						<0.001
≤5	59/379§§	15.6	62/295**	21.0	18.0ΔΔ	0.067
6~7	25/398	6.3	44/318	13.8	9.6	0.001
8	17/220	7.7	27/232	11.6	9.7	0.161
Total	101/997	10.1	133/845	15.7	12.7	<0.001

^§^Chi-Square = 6.89, p = 0.076, trend Chi-Square  = 3.30, p = 0.069.

^§§^Chi-Square = 20.18, p < 0.001, trend Chi-Square = 12.82, p < 0.001.

^*^Chi-Square = 9.53, p = 0.023, trend Chi-Square = 8.92, p = 0.003.

^**^Chi-Square = 10.01, p = 0.007, trend Chi-Square = 9.07, p = 0.003.

^Δ^Chi-Square = 14.27, p = 0.003, trend Chi-Square = 12.17, p = 0.003.

^ΔΔ^Chi-Square = 26.41, p < 0.001, trend Chi-Square = 19.52, p < 0.001.

**Table 3:** Students’ knowledge about burn prevention and first-aid treatments, Zunyi, China.

Questions	Number of choice (N = 1842)	%
What should we pay attention to when using a pressure cooker?		
Open the pot cover after exhausting*	1766	95.9
Open directly	52	2.8
Put your hands on the vent	24	1.3
What should you do if you suffer a burn?		
Spread alcohol or toothpaste	458	24.9
*Cold running water at least 10 minutes*	1235	67.0
Go to hospital directly	149	8.1
Which of the following is true about setting off firecrackers?		
Lighting in your hand	44	2.4
Throw it into a hay after setting it off	85	4.6
Drying the wet firecrackers on the electric stove	29	1.6
*Setting off firecrackers in a spacious place*	1684	91.4
What emergency measures should you take when you are on fire?		
*Take-off clothes right now*	1430	77.6
Rush to hospital	71	3.9
Jump in the pond where you don’t know how deep it is	260	14.1
Don’t know what to do	81	4.4
What should not do when a fire happens?		
Use a wet towel to cover your mouth and nose	189	10.3
Try to send out a rescue signal	78	4.2
*Run into the smog*	1575	85.5
What number should you dial first when a fire happens?		
*Answered correctly (119*)	1682	91.3
Answer incorrectly	160	8.7
What should you do when you find someone who suffered an electric shock?		
Quickly drag him away	74	4.0
*Ask an electrician or adult to pull the plug*	1368	74.3
Pull the wire by hand	400	21.7
What should you do when you see fallen wires on the road?		
Step across	113	6.1
Throw it away with your hand	49	2.7
*Pick it up with dry sticks*	1680	91.2

^*^Italic answers were the correct answers.

**Table 4:** Risk factors for burns among school-aged children in rural areas of Zunyi, China.

Characteristics	Students reported burns, n (%)	All students N (%)	Univariate analysis		Multivariable analysis	
			Crude OR,95% CI		Adjusted OR,95% CI	
Overall	234(12.7)	1842(100.0)	—	—		
Grade level						
3-4	36(15.4)	358(19.4)	1.80	1.19–2.71	1.84	1.20–2.80
5	40(17.1)	422(22.9)	1.92	1.29–2.85	2.00	1.33–2.99
6	66(28.2)	509(27.6)	1.36	0.96–1.99	1.40	0.99–2.00
7	92(39.3)	553(30.0)	1	Ref.		
Gender						
Male	101(43.2)	997(54.1)	1	Ref.		
Female	133(56.8)	845(45.9)	1.66	1.26–2.19	1.70	1.29–2.25
Left-behind child*						
No	140(59.8)	958(52.0)	1	Ref.		
Yes	94(40.2)	884(48.0)	1.44	1.09–1.90	1.35	1.02–1.80
Mother school education						
High school and above	75(32.1)	465(25.2)	1	Ref.		
Secondary school	120(51.3)	889(48.3)	1.23	0.90–1.69	1.19	0.86–1.63
Primary school or below	39(16.7)	488(26.5)	2.21	1.47–3.34	2.43	1.61–3.69
Father school education					—	
High school and above	65(27.8)	420(22.8)	1	Ref.		
Secondary school	116(49.6)	1008(54.7)	1.41	1.02–1.95		
Primary school and below	53(22.6)	414(22.5)	1.25	0.84–1.84		
School boarding					—	
Yes	101(43.2)	632(34.3)	1	Ref.		
No	133(56.8)	1210(65.7)	1.54	1.17–2.04		
Family income (self-reported)					—	
High income	28(12.0)	158(8.6)	0.63	0.40–0.97		
Middle class	170(72.6)	1430(77.6)	1	Ref.		
Poor	36(15.4)	254(13.8)	0.82	0.56–1.20		

^*^whose parents were away in cities, while their children were left behind to be cared for by a single parent or other family members.
